# Electronic Band Structure and Surface States in Dirac Semimetal LaAgSb_2_

**DOI:** 10.3390/ma15207168

**Published:** 2022-10-14

**Authors:** Marcin Rosmus, Natalia Olszowska, Zbigniew Bukowski, Paweł Starowicz, Przemysław Piekarz, Andrzej Ptok

**Affiliations:** 1 Marian Smoluchowski Institute of Physics, Jagiellonian University, Prof. S. Łojasiewicza 11, 30-348 Kraków, Poland; 2 Solaris National Synchrotron Radiation Centre, Jagiellonian University, Czerwone Maki 98, 30-392 Kraków, Poland; 3 Institute of Low Temperature and Structure Research, Polish Academy of Sciences, Okólna 2, 50-422 Wrocław, Poland; 4 Institute of Nuclear Physics, Polish Academy of Sciences, W. E. Radzikowskiego 152, 31-342 Kraków, Poland

**Keywords:** Dirac semimetal, band structure, ARPES, DFT

## Abstract

LaAgSb${}_{2}$ is a Dirac semimetal showing charge density wave (CDW) order. Previous angle-resolved photoemission spectroscopy (ARPES) results suggest the existence of the Dirac-cone-like structure in the vicinity of the Fermi level along the Γ–M direction. This paper is devoted to a complex analysis of the electronic band structure of LaAgSb${}_{2}$ by means of ARPES and theoretical studies within the ab initio method as well as tight binding model formulation. To investigate the possible surface states, we performed the direct DFT slab calculation and the surface Green function calculation for the (001) surface. The appearance of the surface states, which depends strongly on the surface, points to the conclusion that LaSb termination is realized in the cleaved crystals. Moreover, the surface states predicted by our calculations at the Γ and *X* points are found by ARPES. Nodal lines, which exist along the X–R and M–A paths due to crystal symmetry, are also observed experimentally. The calculations reveal other nodal lines, which originate from the vanishing of spin–orbit splitting and are located at the X–M–A–R plane at the Brillouin zone boundary. In addition, we analyze the band structure along the Γ–M path to verify whether Dirac surface states can be expected. Their appearance in this region is not confirmed.

## 1. Introduction

The discovery of topological insulators with a large gap opened a period of intensive studies of this type of topological system [1,2,3]. Due to the intrinsic band inversion, it is possible to realize the topologically protected conducting surface states with linear dispersion, which is called Dirac cones [4,5,6,7,8]. Such surface states paved the way for studying a novel phase of matter [9,10,11].

**Figure 1:**
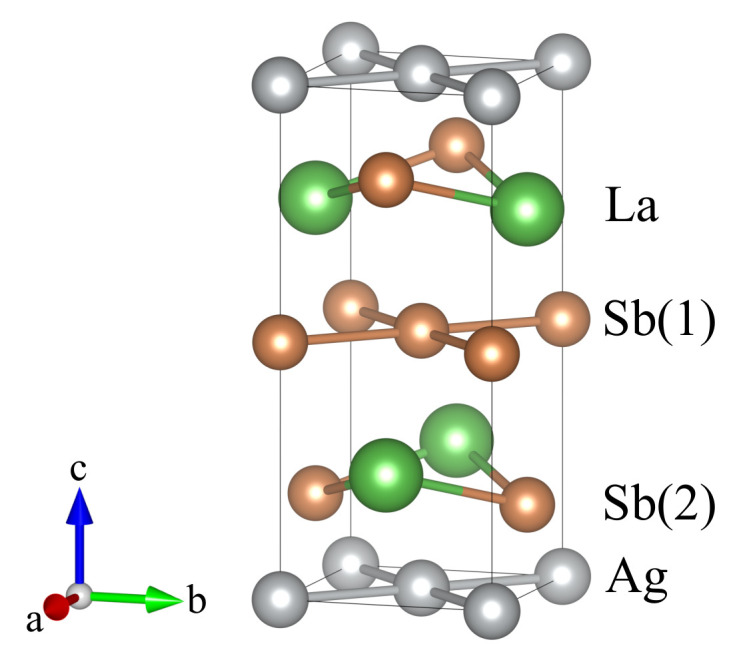
The tetragonal crystal structure of LaAgSb${}_{2}$.

In practice, Dirac cones are observed not only in topological insulators but also in other topological systems. One of the examples, where the existence of these surface states is expected, is the Dirac semimetal LaAgSb${}_{2}$ (Figure 1). It is characterized by the rare and untypical coexistence of the topological phase and the charge density wave (CDW) order [12]. The CDW modulations in LaAgSb${}_{2}$ were found by the X-ray scattering measurements [12] as well as using the pump-probe spectroscopy [13]. In addition, thermal conductivity and ${}^{139}$La nuclear magnetic resonance (NMR) indicate the phase transition [14]. A periodic charge and lattice modulation with the wave vector $q_{1}∼0.026\times \left(2\pi /a\right)$ develops along the *a* direction below the temperature $T_{\mathrm{CDW}}^{1}=207$ K. Further decreasing of the temperature results in an additional CDW ordering below $T_{\mathrm{CDW}}^{2}=186$ K along the *c* direction with $q_{2}∼0.16\times \left(2\pi /c\right)$. The realization of CDW with tiny modulation wave vectors can be associated with the Fermi surface (FS) nesting [15]. Application of the external hydrostatic pressure leads to the suppression of $T_{\mathrm{CDW}}$ and finally to the disappearance of the CDW phases (around 2–3 GPa) [16,17,18]. Similarly, the CDW phase can be destroyed by the chemical doping [17,18].

The existence of the topological phase is indicated by the interesting transport and magnetic properties of LaAgSb${}_{2}$. For example, a large linear magnetoresistance and a positive Hall resistivity were observed [19,20,21]. Similar behavior was also found in the compound with the same structure, namely the topological magnetic system SrMnBi${}_{2}$ [22,23,24]. In that case, the transport behavior is related to the occurrence of the anisotropic Dirac states, where linear energy dispersion originates from the crossing of the *p*-orbital bands in the double-size Bi square net [22]. The first principles study indicated similar properties in the case of LaAgSb${}_{2}$, where the *p* orbitals of Sb atoms create the bands with a nearly linear dispersion [21]. More recently, the angle-resolved photoemission spectroscopy (ARPES) of LaAgSb${}_{2}$ was performed [25].

*Motivation*—The aforementioned ARPES [25] study indicates the existence of several anomalies in the spectral function. In particular, the Dirac-cone-like structure, which originates from the band crossing, was found along the Γ–M direction. In this paper, we performed a systematic and complex study of the electronic band structure of LaAgSb${}_{2}$, using both high-quality ARPES measurements and theoretical analyses. The theoretical study of the electronic band structure was based on the density functional theory (DFT) calculations as well as on the tight binding model. Possible surface states were predicted within the direct DFT calculation for slab geometry and the surface Green function technique for the (001) surface. We show that the realized surface states strongly depend on the termination of the system. Theoretically predicted surface states at Γ and X were confirmed by the ARPES measurements. A presence of nodal lines, which originate either from lattice symmetry or vanishing of spin-orbit splitting, is discussed. Additionally, we analyze the possible Dirac surface states along the Γ–M direction [25]. Our theoretical and experimental study does not confirm the realization of such states along this direction.

This paper is organized as follows. Experimental and theoretical methods are briefly described in Section 2. Next, we presented and discuss our results in Section 3. Finally, Section 4 contains conclusions and a summary.

## 2. Techniques

### 2.1. Sample Preparation

Single crystals of LaAgSb${}_{2}$ were grown by the self-flux technique similar to the method reported in Ref. [20] and also applied for the growth of UCoSb${}_{2}$ crystals [26]. Lanthanum (purity 99.9%), silver (purity 99.99%), and antimony (purity 99.999%) were used as starting materials. The components were weighed in the atomic ratio La:Ag:Sb = 1:1.1:22 and placed in an alumina crucible, which was then sealed in an evacuated silica tube. The ampoule was heated at 1100 ${}^{\circ }$C for 5 h followed by slow cooling (2 ${}^{\circ }$C/h) down to 680 ${}^{\circ }$C. At this temperature, the ampoule was flipped upside down in order to decant still liquid Sb-Ag flux. Next, the ampule was fast cooled to room temperature, and the crucible was transferred to another silica tube, where the rest of Sb was removed from crystals by means of sublimation in high vacuum at 600 ${}^{\circ }$C. Finally, single crystals were mechanically isolated.

The obtained crystals were examined using a scanning electron microscope (SEM), and their chemical composition was determined with energy-dispersive X-ray spectroscopy (EDX) using a standardless procedure. The crystal structure and lattice parameters were determined using powder X-ray diffraction on a sample of the crushed single crystal.

### 2.2. Experimental Details

High-resolution angle-resolved photoemission studies were performed using two ARPES systems, one at Solaris Synchrotron, Kraków, Poland, at URANOS beamline, equipped with a Scienta-Omicron DA30-L electron analyzer (Scienta Omicron, Uppsala, Sweden), and the other at our in-house laboratory with a Scienta R4000 analyzer using He-I radiation (hν=21.218 eV). Samples measured with use of the synchrotron radiation were cleaved in situ in ultrahigh vacuum at room temperature, and the measurements were performed at the temperature of 12 K, while in the in-house laboratory, the samples were cleaved and measured at a temperature of 20 K. The base pressure was below $5\times 10^{-11}$ mbar in both systems. In the case of measurements performed at the synchrotron, the spectra were collected in the vertical and horizontal polarization of the incident light. The ARPES results are presented as the sum of these two scans in order to reduce the matrix element contribution. In order to comprehensively analyze the obtained data, the 2D curvature method [27] was used.

### 2.3. Calculation Details

The DFT calculations were performed within the projector augmented-wave (PAW) method [28] using the Vienna Ab initio Simulation Package (vasp) [29,30,31]. The exchange-correlation potential was obtained by the generalized gradient approximation (GGA) in the form proposed by Perdew, Burke, and Enzerhof (PBE) [32]. We also investigated the impact of the spin–orbit coupling (SOC) [33] on the electronic structure.

The optimization of both the structural parameters and the electronic structure was performed using a 20×20×10 Monkhorst–Pack **k**-grid [34]. The energy cut-off for the plane-wave expansion was equal to 520 eV. The structures were relaxed using the conjugate gradient technique with the energy convergence criteria set at $10^{-8}$ eV and $10^{-6}$ eV for the electronic and ionic iterations, respectively. Next, the optimized structure was used to construct the tight binding model in the maximally localized Wannier orbitals (cf. Appendix A). The surface Green function for a semi-infinite system [35] for study of the surface states was calculated using WannierTools (version 2.5.1) [36].

## 3. Results and Discussion

### 3.1. Crystal Structure

LaAgSb${}_{2}$ crystallizes in the tetragonal ZrCuSi${}_{2}$-type lattice [37] with the layered structure *P4/nmm* (space group 129) presented in Figure 1. The obtained experimental crystals are rectangular and plate-like with a crystallographic *c* axis perpendicular to the plane. The chemical composition of the crystals, determined from the EDX data, corresponded well to the ideal formula LaAgSb${}_{2}$. The lattice parameters determined from XRD (a=b=4.390 Å and c=10.84 Å) are in good agreement with those reported in the literature (a=b=4.359 Å, and c=10.787 Å [38,39]). In our calculations, the lattice constants were found as a=b=4.43974 Å, and c=10.89658 Å, which are in good agreement with the experimental results. The Wyckoff positions of atoms are La: 2c (14,14,0.73994), Ag: 2a (34,14,0), Sb(1): 2b (34,14,12), and Sb(2): 2c (14,14,0.17137). The Sb(1) atoms form a two-dimensional square lattice, while the Sb(2) atoms coordinated by La atoms form a LaSb(2) double layer characterized by the glide symmetry. The Ag atoms reside between two LaSb(2) layers. The layered structure can be viewed as a sequential stacking of Sb(1)–LaSb(2)–Ag–LaSb(2)-Sb(1) layers along the *c* axis (cf. Figure 1).

### 3.2. Band Structure and Fermi Surface

**Figure 2:**
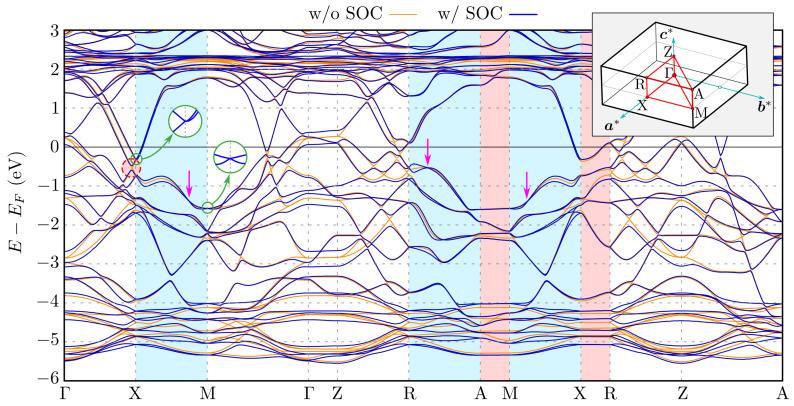
The bulk band structure in the absence and presence of the spin–orbit coupling (orange and blue lines, respectively). X–M and R–A paths, where nodal lines can be realized due to vanishing spin–orbit splitting, are marked with blue background. Similarly, X–R and A–M paths are highlighted with red background, where nodal lines with fourfold degeneration exist due to the *P4/nmm* symmetry. Two circular insets highlight the bulk Dirac cones at the X and M points. The top right inset presents the Brillouin zone of the tetragonal structure *P4/nmm* and its high-symmetry points [42].

The ab inito (DFT) calculated bulk band structure is presented in Figure 2, where we show the comparison of the results in the absence and presence of the spin–orbit coupling (orange and blue lines, respectively). They are in agreement with the previous study [21,40,41]. The unoccupied La 5*f* orbitals are located above the Fermi level (around 2 eV). The 5*p* orbitals are responsible for the emergence of the nearly linear band crossing the Fermi level [21,41]. These bands are associated with the several places where band crossing exists. These points arise due to the folding of the dispersion relation of the *p*-orbitals in Ag and Sb nets. Including the spin–orbit coupling removes the degeneracy of these points.

Around the X point, we can observe the influence of the SOC on the electronic band structure. Along the Γ–X direction without the SOC, there are four different irreducible representations of bands, and the crossing of bands exists [43]. The presence of the SOC allows for only one irreducible representation and causes band splitting as well as gap opening close to the X point (marked by the dashed red circle in Figure 2). Additionally, the energy difference between two Dirac-like cones at the X point (first zoom inside left inset in Figure 2 and second non-shown below them) typically strongly depends on c/a [43]. The described modification originates mostly from folding of the band structures of Ag and Sb square nets [44,45], which is due to the realization of their double unit cell, i.e., $\sqrt{2}\times \sqrt{2}\times 1$ cell, within the LaAgSb${}_{2}$ cell (cf. Figure S1 in the Supplemental Material (SM), which contains more additional theoretical results).

*Highly degenerated nodal lines*—Including SOC leads to lifting of the band degeneracy, while the time reversal symmetry is still preserved due to the absence of the magnetic order.

Additionally, the space group *P4/nmm* guarantees the existence of the nonsymmorphic glide symmetry [45,46]. Assembling these symmetries with the three-dimensional (3D) inversion symmetry has a consequence in the form of the bulk band structure [47]. Along the high symmetry directions, all the bands at the X and M points are doubly degenerated by the glide mirror symmetry, forming Dirac points (two left insets in Figure 2 and Figure S2 in the Supplementary Materials). Extending to 3D [47], all lines along $k_{z}$ containing X and M points (i.e., X–R and M–A) form degenerated lines called nodal lines (NLs). For example, in Figure S3, in the Supplementary Materials, we show forming of the NL along the X–R direction by the crossing of two bands. NLs are formed from the crossing of two Dirac-like bands. A similar situation is observed in the other Dirac semimetals [43,46,48,49,50,51,52,53,54,55,56,57,58].

**Figure 3:**
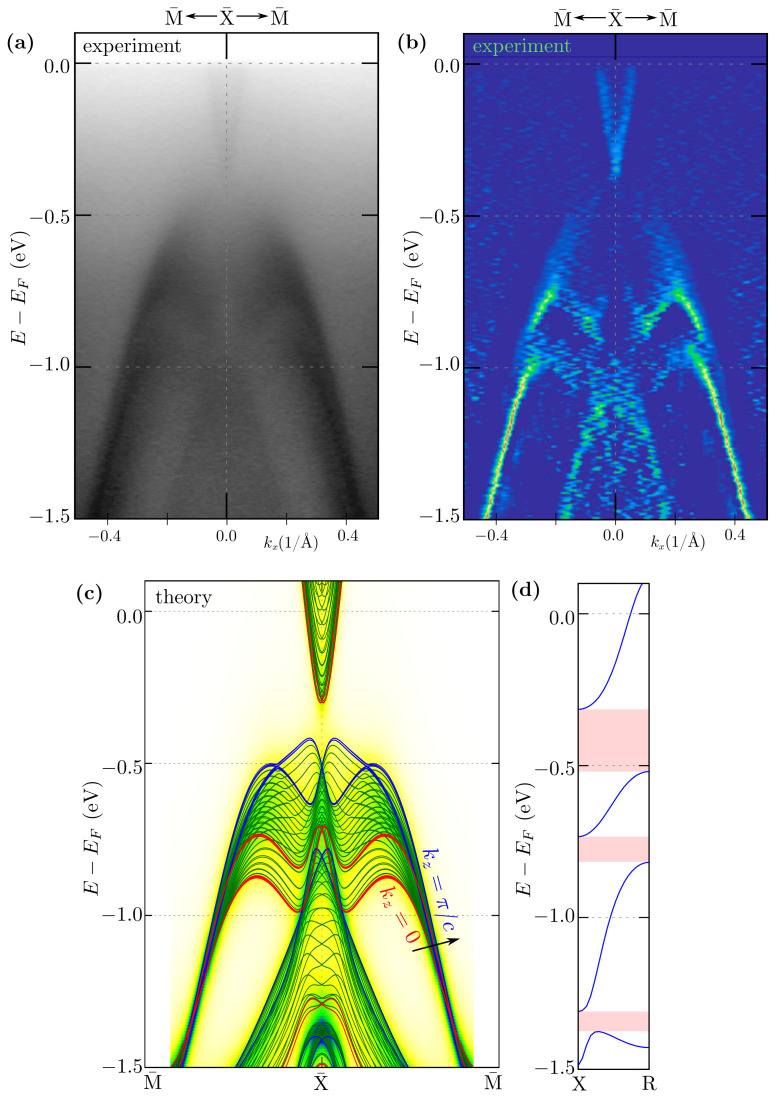
Comparison of the ARPES spectra (**a**) and corresponding 2D curvature (**b**) with the theoretical calculation: spectral function (background) and projected bulk bands (lines) (**c**). The results are presented along the $\bar{X}$–$\bar{M}$ direction, around the $\bar{X}$ point. The experimental data have been collected with photon energy hν=66 eV at a temperature of 12 K. Panel (**d**) presents the energy range of the nodal lines in the bulk band structure along the X–R path (red areas mark the band gap between the nodal lines).

**Figure 4:**
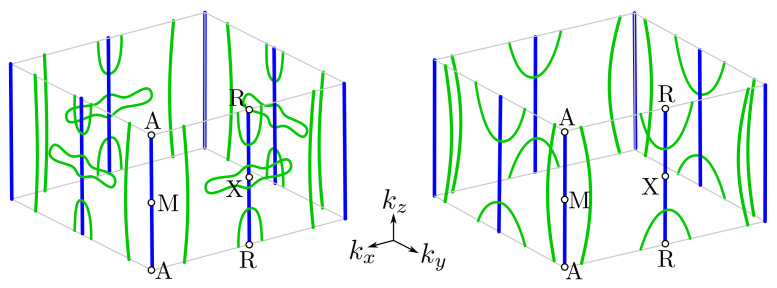
Location of nodal lines in **k**-space with respect to the first Brillouin zone (gray box). Actual, variable binding energy is not indicated. Blue solid lines are realized due to the system symmetry (sample range of energies are presented in Figure 3d). Similarly, green solid curves are realized as a consequence of the spin–orbit splitting vanishing at the Brillouin zone boundary (in X–R–A–M planes). Green lines are located at binding energies in ranges from −1 to −2 eV, and from −0.5 to −1.0 eV, for the left and right panels, respectively.

In a general case, NLs can be observed experimentally, in the form of the bands crossing [59]. Its binding energy varies with $k_{z}$, which can be visualized by changing photon energy. In our case, NLs should be visible in the form of the band crossing at the $\bar{X}$ points, where the bulk NLs from the X–R path are projected (Figure 3). Theoretical investigation of the bulk NLs recovers the existence of several gaps between them (marked by red areas in Figure 3d). For instance, NLs inside the upper band fill approximately the energy range from −0.7 to −0.5 eV below the Fermi level. In this range of energies, several $k_{z}$-depending band crossings are visible (see Figure 3c) Unfortunately, the exact band crossing is not well resolved in the experiment (top panels on Figure 3). However, the depletion of spectral intensity in the experiment is observed for binding energies around ∼−0.4 eV corresponding to the gaps at $\bar{X}$. Additionally, we observed excellent agreement of the experimental results with the theoretical ones (cf. top and bottom bands in Figure 3). These nodal lines are shown as blue lines in Figure 4, which presents the location of the nodal lines in the **k**-space.

The SOC slightly lifts the degeneracy along the X–M and R–A lines. However, the highly degenerated nodal lines (e.g., places marked by pink arrows in Figure 2) also can be found in this direction, which is due to vanishing of the spin–orbit splitting for some k. These fourfold degenerated Dirac points lead to the emergence of an additional NL at the boundary of the Brillouin zone (in the X–M–A–R planes). The NLs are presented schematically in Figure 4 by green lines (we present NLs only for eight bands near the Fermi level). As we can see, some of the NLs create closed contours along $k_{z}$ located near the A–M edge of the Brillouin zone. Contrary to this, other NLs form closed ellipsoidal contours around an R high-symmetry point.

**Figure 5:**
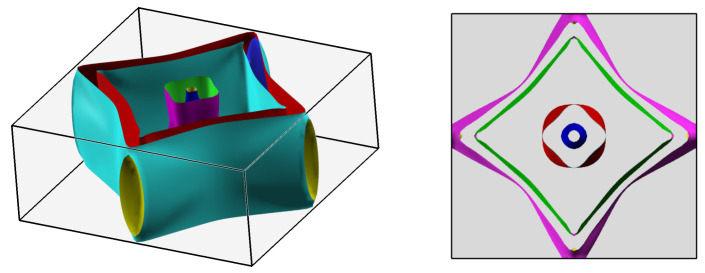
Three-dimensional and top view (**left** and **right** panels, respectively) of the Fermi surface of LaAgSb${}_{2}$ in the absence of the spin–orbit coupling.

*Fermi surface*—The previous studies showed that the FS in the absence of the SOC (Figure 5) is composed of four bands [15,19,40,60]: two cylindrical-like pockets along the Γ–Z direction and two pockets with diamond-like shapes connecting the X points of the first Brillouin zone (separate pockets are shown in Figure S4 in the Supplementary Materials). Analyses of the band structure of the separate layers of LaAgSb${}_{2}$, i.e., Sb and Ag square nets, and LaSb double-layer (cf. Figure S1 in the Supplementary Materials), clearly show that the pockets around the Γ–Z direction are associated with the LaSb double layer (mostly 5*d* orbitals of La and 5*p* orbitals of Sb). Similarly, the quasi-two dimensional Sb and Ag square nets create diamond-like pockets (5*s* and 4*d* orbitals of Ag and 5*p* orbitals of Sb).

**Figure 6:**
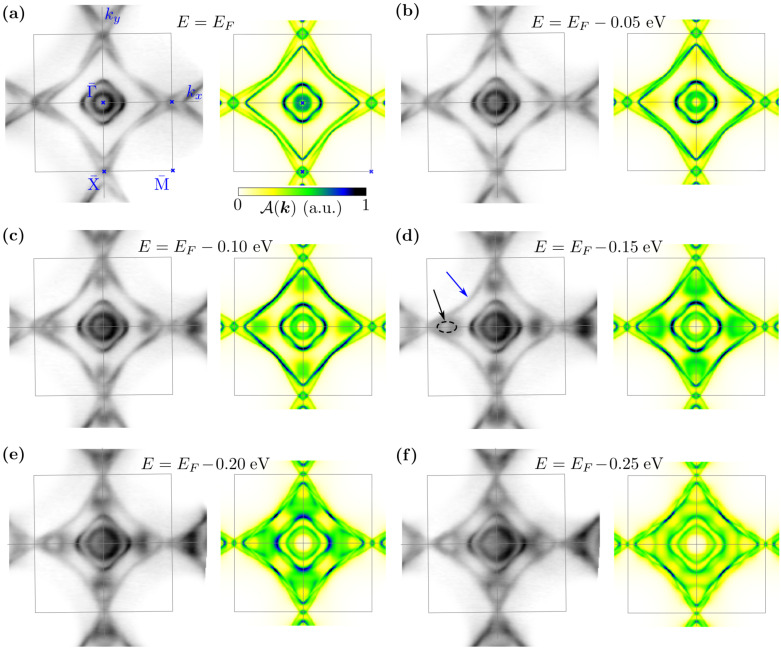
Comparison of the ARPES spectra obtained with a photon energy of 66 eV at a temperature of 12 K (**left panels**) with the theoretical surface spectral function (**right panels**) for selected binding energies *E* below the Fermi level (as labeled). The gray square denotes the Brillouin zone. The black arrow and the dashed outline indicate the additional branch explained by the spectral function.

The isoenergetic study of the spectral function clearly shows that the characteristic shape of the FS is observed experimentally. A comparison of the experimental and theoretical results is presented in Figure 6 in the left and right panels, respectively. First, the ARPES result for the energy corresponding to the Fermi level $E_{F}$ (Figure 6a) is clearly reproduced by the theoretical projected Fermi surface. Similarly as in the bulk system (Figure 5), we can find two pockets centered at the $\bar{\Gamma }$ point and two diamond-like pockets connecting the $\bar{X}$ points. Our detection of the pocket around the $\bar{\Gamma }$ point is in agreement with the previous measurements for this material [61]. In both the experimental and theoretical results, we can find a few characteristic features. The internal $\bar{\Gamma }$-centered pocket shows weak $k_{z}$-dependence (theoretical spectral weight of this pocket has an approximately constant intensity). Contrary to this, the external $\bar{\Gamma }$-centered pocket shows stronger $k_{z}$-dependence in the $\bar{\Gamma }$–$\bar{M}$ direction than in $\bar{\Gamma }$–$\bar{X}$—this is well visible in the form of the increasing theoretical spectral weight along the second direction. The situation looks similar in the case of the diamond-like pockets. Here, we must mention that a similar diamond-like FS projection is observed also in the other Dirac semimetals within this family, e.g., LaCuSb${}_{2}$ [62], YbMnBi${}_{2}$ [63] and YbMnSb${}_{2}$ [64], or SrMnBi${}_{2}$ and CaMnBi${}_{2}$ [65], as well as square-net materials [51], e.g., ZrSiTe [43], ZeSiS [52,55,56], ZrSnTe [57], ZrGeTe [58], HfSiS [49,50], ZrSiS [48,49], SmSbTe [54], LaSbTe [46], or NbGeSb [66].

The characteristic features for $E=E_{F}$ are conserved also below $E_{F}$. However, going below $E=E_{F}$, we can observe a few important behaviors. First, we notice the *E* dependence of two branches forming two diamond-like pockets of the FS (marked with the blue arrow at Figure 6d). Under decreasing *E*, we find the hourglass shape by the band branches (which will be discussed more precisely in the next paragraphs). Secondly, around $E=E_{F}-0.1$ eV, we can observe the appearance of the parabolic-like branch in the band structure near the $\bar{X}$ point. For lower *E*, this branch is well visible in the form of a circular shape in the spectral function (marked by black dashed lines and the black arrow in Figure 6d). For $E=E_{F}-0.25$ eV, this branch is linked to the diamond-like shape. It is worth mentioning that in the whole range of *E*, the theoretical calculations are comparable with the experimental ARPES results (cf. left and right panels in Figure 6a–f), which indicate the weak role of the electron correlations in this system in the considered energy scale.

With decreasing *E*, modification of the FS pocket around the $\bar{X}$ point can be observed (Figure 6). This pocket is formed by a common part of two diamond-like pockets from the two nearest Brillouin zones. The change in the size of these pockets observed for the decreasing binding energy leads to the disappearance of the pocket crossing the border of the Brillouin zone (cf. panels from Figure 6a–f). Indeed, we can reveal this behavior carefully analyzing both experimental data and the theoretical spectral function along the $\bar{X}$–$\bar{M}$ direction, i.e., at the Brillouin zone boundary (Figure 3c). The electron pocket crossing the Brillouin zone boundary vanishes around the energy ∼0.35 eV below the Fermi level. Theoretical analyses show that the upper (electron) (above ∼−0.35 eV) and lower (hole) bands (below ∼−0.5 eV) are separated at the $\bar{X}$ point by the ∼0.15 eV band gap. The lower bands create a characteristic double parabolic structure corresponding to experimental results (Figure 3a).

### 3.3. Surface States within Direct DFT Slab Calculations

**Figure 7:**
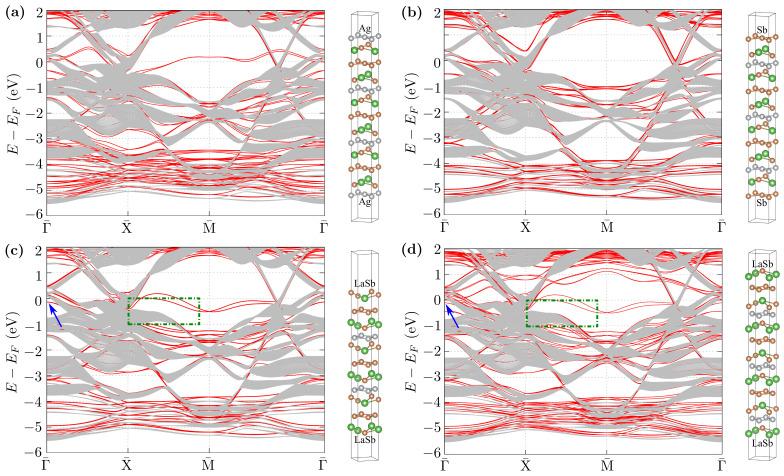
Band structures in slab geometry (with corresponding structures presented on right-hand side) dependent on the surface termination of Ag (**a**) and Sb (**b**) square nets and two possible terminations of the LaSb layer—conserving (**c**) and breaking (**d**) glide symmetry. Red lines denote the slab band structure from the DFT calculations, while gray lines denote the projected bulk band structure from tight binding models calculations (for eighty $k_{z}$ different momenta). Blue arrows indicate the surface states below the Fermi level at the Γ point. The green dotted-dashed line in (**c**,**d**) shows the area presented in Figure 8.

To evaluate the data described in the previous section, we performed the DFT calculation for slab geometry with different terminations (Figure 7), namely square net of Ag atoms (Figure 7a), square net of Sb atoms (Figure 7b), and two nonequivalent LaSb layers shown in Figure 7c,d. The structural models used in the DFT calculations are presented at the right side of the obtained band structures (Figure 7). Each time, ∼10 Å of vacuum is included.

To extract the surface states, we compare calculated band structures of slabs (solid red lines) with the bulk band structures (solid gray lines) projected onto the 2D reduced Brillouin zone. In the case of the bulk bands, around the $\bar{M}$ point, there exists a relatively large “gap” between continuum states which allows extracting the surface states in a relatively simple way. Indeed, in this region, the surface states are well visible. For three cases of chosen terminations, the surface states crossing the Fermi level are well visible around the M point, where they form Dirac cones located either above (Figure 7a) or below (Figure 7c,d) $E_{F}$. In the case of Sb termination (Figure 7b), the surface states are deep below the Fermi level (around −1 eV). Similar states can be observed experimentally in some materials, such as NbGeSb [66]. The absence of such Dirac surface states at M in the photoemission data would suggest the realization of the Sb or Ag square net termination. Similarly, only LaSb termination allows the realization of the surface states near $E_{F}$ in the region of Γ point, as shown by blue arrows in Figure 7c,d. Nevertheless, the most interesting range of energies (around the Fermi level) corresponding to the paths $\bar{\Gamma }$–$\bar{X}$ and $\bar{M}$–$\bar{\Gamma }$ is filled by the bulk states. Contrary, e.g., to the topological insulators [4,5,6,7,8], the extraction of separate surface states can be hard or even impossible within the existing ARPES data due to the presence of bulk bands.

*Role of surface termination*—As we can see from the comparison of the results for different surface terminations, there exists a strong dependence of the realized surface states on a type of termination (cf. panels on Figure 7). Such behavior was reported before, e.g., in the case of Weyl semimetals [67]. At the same time, in each case, nearly linear bulk bands crossing the Fermi level are insensitive to the modification of termination. The same bulk bands play the most important role in the described earlier ARPES data due to the weak $k_{z}$ dependence of the corresponding dispersion relations. To conclude this part, we can propose that the sample studied experimentally was terminated by the LaSb layer. However, the discussed surface states should be absent if the surface termination resulting from cleaving is different.

From a theoretical point of view, the most favorable type of termination can be predicted by calculation of the binding energy within the DFT framework. To find this type of prediction, we calculated the binding energy ΔE between the adjacent layers: $\Delta E=E_{A+B}-E_{A}-E_{B}$, where $E_{A+B}$, $E_{A}$, and $E_{B}$ denote the energy of products (A + B) and individual reactants (A and B), respectively. In practice, $E_{A}+E_{B}$ can be calculated when the last layer (B) is ∼10 Å above substrate (A) with respect to the investigated surface (A + B). The results of our calculation are collected in Table 1. As we can see, independently of the surface termination, the binding energy is always negative; i.e., there is no unique preferred termination.

### 3.4. Surface States around $\bar{\Gamma }$ and $\bar{X}$

**Figure 8:**
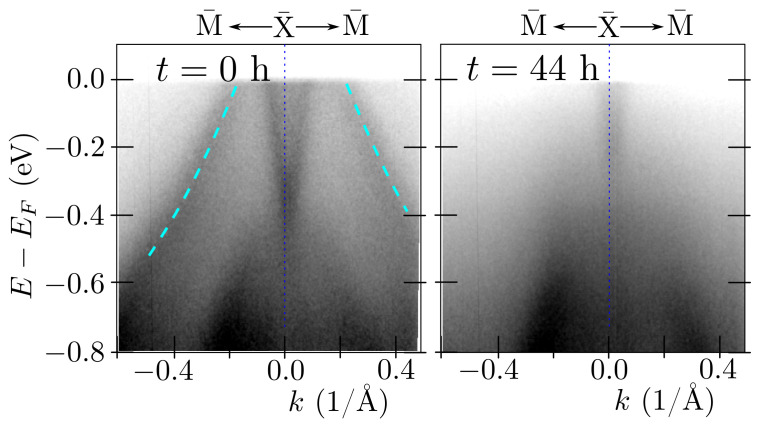
The ARPES results obtained around the $\bar{X}$ point presenting the realized surface states. The panels present spectra collected at different time points following the sample cleaving. Cyan dashed lines highlight the surface state. The measurements have been realized with He-I (hν=21.218 eV) radiation for the sample cleaved and measured at a temperature of 20 K.

ARPES measurements, which visualize surface states better, were performed directly following the cleaving at 20 K. For this case, the ARPES spectra obtained along the $\bar{X}$–$\bar{M}$ direction, around the $\bar{X}$ point are presented in Figure 8. Now, we observe an additional band in the range of energies, where bulk states are not realized. This additional band disappeared much quicker compared to the rest of the structure (cf. left and right panels, presenting results at a different time following the cleaving of the crystal). What is important is that the decay time was different in different vacuum systems, namely from several minutes to tens of hours. This is a consequence of surface contamination by impurities from the environment. All this makes a strong suggestion that the described band is of surface origin. By comparing these results with the theoretical prediction, we can conclude that the LaSb termination is realized for this sample (cf. Figure 7c,d). A quick look at the crystal structure (Figure 1) leads to the conclusion that if LaSb termination results from cleaving, a neighboring layer of either Ag or Sb should also form another termination. The absence of the characteristic band of Ag termination in the ARPES spectra at the M point (Figure 7a) indicates that rather not Ag but Sb termination may be present as well. However, we do not have any direct proof of this assumption, and it is also not clear if another termination is ordered to form surface states.

**Figure 9:**
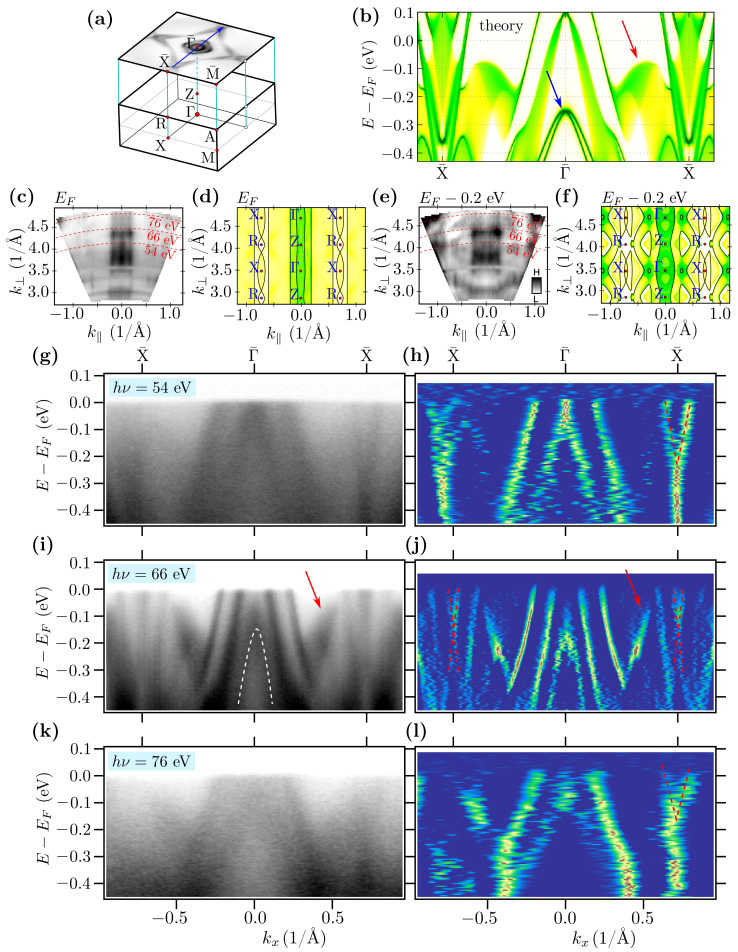
Cartoon representation of the bulk first Brillouin zone projection into the surface Brillouin zone (**a**). Theoretical surface spectral function along the $\bar{X}$–$\bar{\Gamma }$–$\bar{X}$ direction (**b**). Panels from (**c**–**f**) present intensity plots for constant energy planes at the Fermi level $E_{F}$ and energy $E_{F}-0.2$ eV (as labeled). The ARPES spectra (**c**,**e**) were collected in the photon energy range from 20 to 80 eV. The calculated band structure (solid black lines) along with the spectral function (background color scale) is shown in (**d**,**f**). Here, the intensity maps is a function of $k_{⊥}=k_{z}$ and $k_{\|}=k_{x}$ (perpendicular and parallel to the surface component of the wave vector). Panels (**g**–**l**) present experimentally obtained spectra and their 2D curvature (left and right panels, respectively) for different photon energies (as labeled). The red arrows in (**b**,**i**,**j**) indicate the position of additional structure in the spectra that can be explained by the projection of the bulk band on the surface Brillouin zone. The blue arrow in (**b**) and white dashed parabola in (**i**) indicate the surface state. The red dashed lines in (**h**,**j**,**l**) refer to the position of the band crossing of the band forming the nodal line. The measurements were made at the temperature of 12 K.

The band structure along the $\bar{\Gamma }$–$\bar{X}$ direction is presented in Figure 9. As it can be seen, in the experimental spectra, we can well separate visible bands. The most intensive lines demonstrate the shape of the projected FS in a form of a pair of cylindrical pockets centered at $\bar{\Gamma }$ and a pair of diamond-like pockets (cf. Figure 5a). Additionally, exactly at the Γ point, around −0.2 eV, we can see the surface states in a form of the parabolic-like band (highlighted by a dashed white line in Figure 9i). Observation of this surface state is in agreement with the direct DFT calculations (cf. discussion in Section 3.3) and surface Green function calculations (surface state is marked by blue arrow in Figure 9b).

Additionally, examination of the band structure along the $\bar{\Gamma }$–$\bar{X}$ direction uncovers also other features of the band structure discussed earlier. The NLs are visible as a crossing of the bands at the $\bar{X}$ point. This crossing is visible independently of the energy of photons used during the ARPES experiment (red dashed lines on right panels in Figure 9). ARPES scans with variable photon energy yield constant energy intensity maps as a function of $k_{⊥}=k_{z}$ and $k_{\|}=k_{x}$ (perpendicular and parallel to the surface component of the wave vector) in the Γ–X–R–Z plane, and the data are compared to the calculated spectral function (see from Figure 9c–f). The data are shown for $E_{F}$ (cf. Figure 9c,d) and −0.2 eV below $E_{F}$ (cf. Figure 9e,f). The spectra have been collected for photon energies in the range from 20 to 84 eV, and $k_{⊥}$ was obtained with the assumed inner potential of $V_{0}=15$ eV. The comparison of the spectra and theory may indicate the actual assignment of high symmetry points as proposed in Figure 9c–f. Taking into account this last result, as well as the excellent agreement of experimental and theoretical data (e.g., Figure 3), we conclude that the hν=66 eV corresponds to the $k_{z}$ close to the Γ–X–M plane. Indeed, the existence of the band crossing independently on $k_{⊥}$ confirms the realization of NL at $\bar{X}$ (which has been discussed earlier).

Moreover, around −0.1 eV, we can observe a blurred shape (marked by the red arrow in Figure 9i). The theoretical study clearly shows that the additional structure in the spectra can be explained by the projection of the bulk band on the surface Brillouin zone. Indeed, the calculated spectral function confirms this explanation (cf. red arrow in Figure 9b). This structure was also observed in the form of a cylindrical shape in the isoenergetic cuts (e.g., marked by a black arrow in Figure 6d).

### 3.5. Surface States along $\bar{\Gamma }$-$\bar{M}$

**Figure 10:**
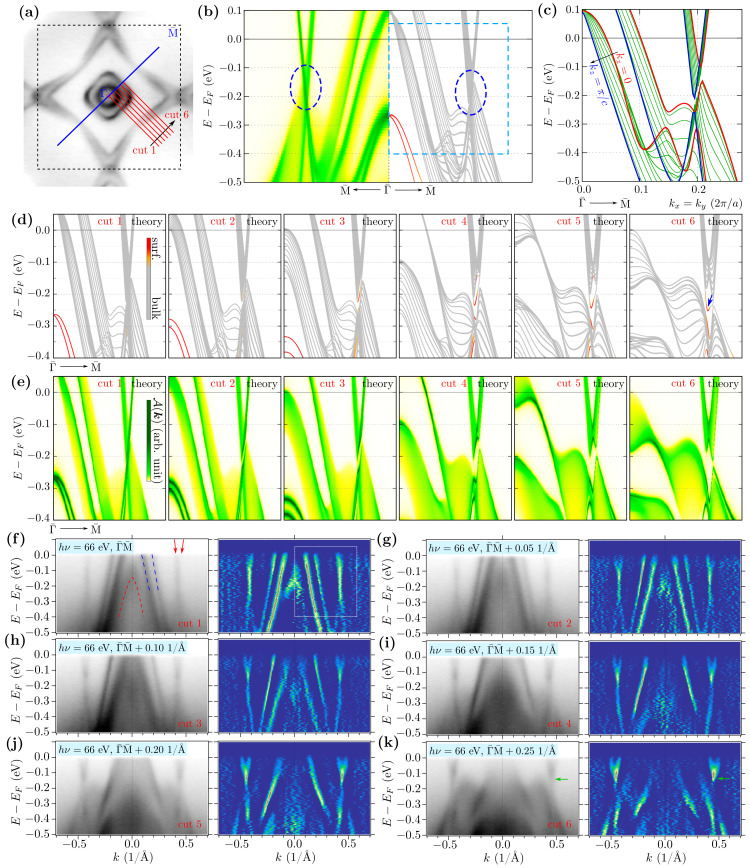
The electronic band structure along a series of paths presented in (**a**). Theoretical spectral function (left) and band structure (right) along the $\bar{\Gamma }$–$\bar{M}$ path for a finite system (**b**) and related bulk band structure (**c**). Panels (**d**) present band structures calculated for the slab geometry including both LaSb and Sb terminations along different cuts shown in (**a**). Bands with red (gray) color correspond to the surface (bulk) states. Theoretical prediction of the spectral function within the surface Green’s function calculation for LaSb termination along different cuts from (**a**) is presented in (**d**). Panels (**d**,**e**) correspond to the range of parameters marked by boxes in (**b**,**f**). Panels (**f**–**k**) show the experimental spectra recorded with the ARPES measurements with hν=66 eV at a temperature of 12 K (left panels) and their 2D curvatures (right panels) along different cuts from (**a**). The blue dashed lines in (**f**) indicate the bands forming central pockets of the FSs, while the red dashed line refers to the surface state. The red arrows in (**f**) indicate the position of the band corresponding to the diamond-like part of the FS, and the green arrows in (**k**) point to the bottom of the band above the energy gap related to the blue arrow on cut 6, i.e., panel (**d**).

In the bulk band structure, there exist several places where the SOC leads to opening of the gap between initially crossed bands. Many of them are located along the Γ–M and Z–R paths with energies close to the Fermi level. The previous study of Shi et al. [25] suggested the realization of the Dirac cone-like structure in the vicinity of the Fermi level formed by the crossing of two linear energy bands. In this section, we present a complex study of the electronic band structure, based on theoretical and experimental results, along different cuts parallel to Γ–M (Figure 10).

The surface Green function and slab band structure are presented in Figure 10b (left and right half, respectively). The crossing of the linear bands around −0.15 eV is well visible in both results (marked by blue dashed ellipses). Projection of the bulk electronic bands on the surface Brillouin zone directly presents the $k_{z}$ dependence of separate bands (Figure 10c). Independently of $k_{z}$, the direct gap is realized in the bulk (around $k_{x}=k_{y}≃0.2\times 2\pi /a$). The value of this gap can be estimated as ∼5 meV, which can be seen in a high-resolution ARPES experiment. Additionally, this gap changes its value for directions away from the Γ–M path (cf. Figure S6 in the Supplementary Materials). Moreover, the bulk gap is shifted from energy −0.125 to −0.225 eV below the Fermi level.

Direct analysis of the band in slab geometry (including both LaSb and Sb terminations) is presented in Figure 10d, where the color of line denotes the contribution of the surface or bulk states (red and gray color, respectively). As previously, the surface states at the $\bar{\Gamma }$ point are visible, which is realized in a system with the LaSb termination. The other surface state (marked by the blue arrow) is realized by the second surface of the slab, which is terminated by the Sb square net. Here, we should have in mind that only LaSb termination was directly indicated by ARPES for our system. Indeed, the surface Green function calculated for the LaSb termination (Figure 10e) correctly reproduces the surface state found at the $\bar{\Gamma }$ point. At the same time, the other surface state is not observed. The corresponding experimental results for different cuts are presented in panels from Figure 10f–k. Firstly, we observe the disappearance of the surface state at the $\bar{\Gamma }$ point (red dashed line in Figure 10f) while moving away from the Γ–M direction. Secondly, we observe a strong momentum-dependence of the bands forming central pockets of the FSs (two blue dashed lines in Figure 10f). For instance, at the cut 4, which crosses only the external $\bar{\Gamma }$-centered FS, we clearly see the shift of the internal branches to lower energies. Two branches corresponding to the diamond-like part of the FS (marked by two red arrows in Figure 10f) also yield slightly different dispersions. These two branches should play a role in the realization of the discussed surface state. Directly along the $\bar{\Gamma }$–M direction (cut 1), a cross-like structure is visible. Away from this cut, the open gap becomes more visible, where the bottom of the upper branches is visible (marked by a green arrow in Figure 10k).

The surface Green function correctly reproduces the experimental results. In both cases, the surface state closing the gap is not visible.

## 4. Summary

In this paper, we have performed the systematic high-resolution ARPES measurements on LaAgSb${}_{2}$ covering a large area of the Brillouin zone. The presented experimental results were supported by the theoretical analyses, within the ab initio (DFT) calculations, as well as by the tight binding model formulation. Both theoretical techniques allow for excellent reproduction of the experimentally observed band structure of LaAgSb${}_{2}$ (cf. Section 3.2). Nodal lines related to the symmetry of the system are shown in calculations, and the corresponding ARPES spectra are obtained. Moreover, analyses of the band structure uncover the highly degenerated nodal lines, the existence of which is not a consequence of the *P4/nmm* symmetry. These nodal lines occur due to vanishing the spin–orbit splitting, and they are located at the boundary of the Brillouin zone.

Direct studies of the system with slab geometry indicate a possibility of realization of the surface states in this compound (Section 3.3). We conclude that the appearance of the surface states strongly depends on the termination. For instance, the surface states can be realized in the form of well visible separate parabolic-like bands when the sample is not terminated by the Sb layer. Indeed, our experimental data confirm this theoretical prediction (Section 3.4). We find the surface states at the Γ and X points in the form of parabolic-like bands. However, this state disappears relatively fast due to the adsorption of surface contamination. Nevertheless, the observed surface states at the Γ and X points indicate the LaSb termination of the studied sample.

Finally, we verified if the Dirac surface states can be found along the Γ–M direction. Careful analysis of this problem was presented in Section 3.5. The theoretical examination of the band structure clearly shows that the Dirac surface state can appear only at the Sb square net termination, in contrast to the LaSb termination found in our samples. As a consequence, the Dirac surface states along Γ–M are not observed. Similar results can be obtained for the surface Green function analyses, where the surface state is also absent.

## Figures and Tables

**Table 1:** The comparison of the binding energies ΔE (as a difference between the energy of the surface $E_{A+B}$, and energy of individual reactants $E_{A}+E_{B}$) for different terminations (as labeled).

Termination		$E_{A+B}$	$E_{A}+E_{B}$	ΔE
		(eV)	(eV)	(eV)
Ag term	[Figure 7a]	−99.00	−96.86	−2.14
Sb term	[Figure 7b]	−99.00	−96.67	−2.33
LaSb term	[Figure 7c,d]	−84.36	−82.55	−1.81

**Figure A1:**
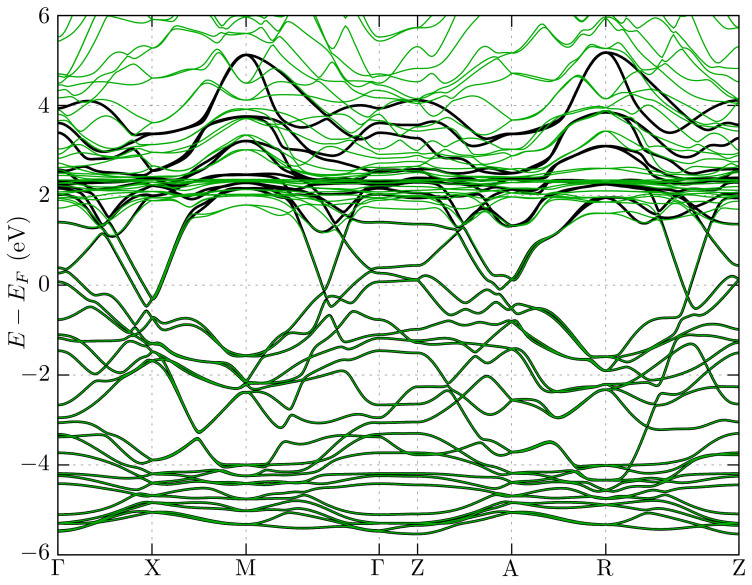
The comparison of the band structure obtained from the DFT calculations (green lines) and tight binding model (black lines).

**Figure A2:**
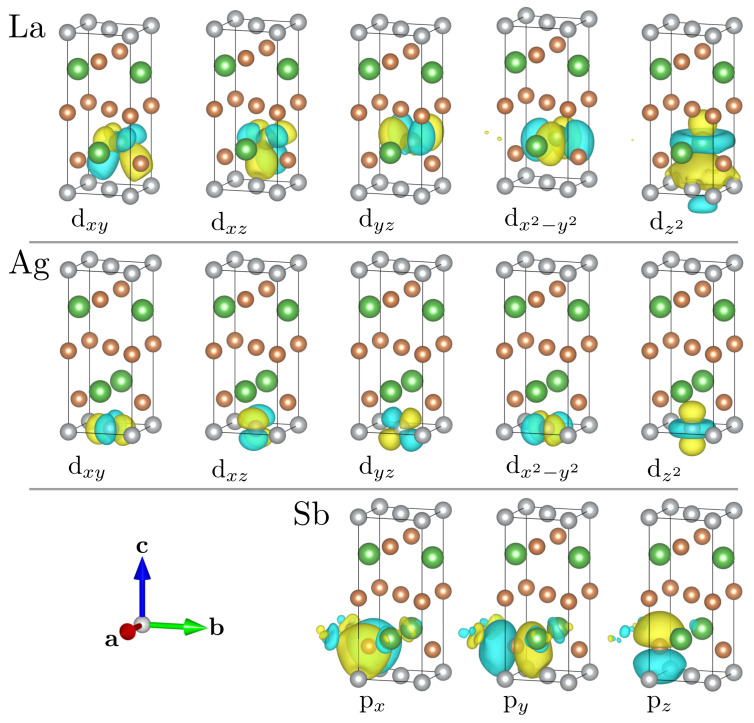
The final form of the maximally localized Wannier orbitals, found during procedure of the DFT band structure wannierization. Groups from top to bottom correspond to *d*-like orbitals of Ag and La, and *p*-like orbitals of Sb, as labeled.

## Data Availability

Not applicable.

## Supplementary Materials

The following supporting information can be downloaded at: https://www.mdpi.com/article/10.3390/ma15207168/s1, Figure S1: the bulk electronic band structure related to layers forming LaAgSb${}_{2}$ structure; Figure S2: the Dirac points at X and M, Figure S3: nodal lines formed along X–R line, Figure S4: Fermi surface branches shown separately, Figure S5: comparison of the band structures in *P4/mmm* (not realized in reality, SG:123) and *P4/nmm* (SG:129) showing impact of the mirror symmetry and glide symmetry, Figure S6: the bulk electronic band structure along Γ–M path for $k_{z}=0$ and $k_{z}=\pi /c$.

## Appendix A. Tight Binding Model in Maximally Localized Wannier Orbitals Basis

Using results of the DFT calculation for electronic band structure we can find the tight binding model in the basis of the maximally localized Wannier orbitals [70,71,72]. It can be performed via the Wannier90 software (version 3.0.0) [73,74,75]. As a result of this we can find the parameters of the tight binding Hamiltonian of free electrons in the form:

(A1) $$\begin{array}{c} H=\sum_{R,R^{\prime }}\sum_{\mu ,\mu^{\prime }}\sum_{\sigma ,\sigma^{\prime }}t_{R\mu \sigma ,R^{\prime }\mu^{\prime }\sigma^{\prime }}c_{R\mu \sigma }^{†}c_{R^{\prime }\mu^{\prime }\sigma^{\prime }}, \end{array}$$

where $c_{R\mu \sigma }^{†}$ ($c_{R\mu \sigma }$) denotes the creation (annihilation) operator of the electron with spin σ at μ orbital localized on atom in R position. Here $t_{R\mu \sigma ,R^{\prime }\mu^{\prime }\sigma^{\prime }}$ for $\sigma =\sigma^{\prime }$ ($\sigma \neq \sigma^{\prime }$) denotes the spin conserved (spin flip) hopping integrals between orbitals $O_{1}=\left(R\mu \right)$ and $O_{2}=\left(R^{\prime }\mu^{\prime }\right)$. We should notice that the spin flip hopping part corresponds to the SOC, i.e., mixing of the different type of spin subspaces. From this, the normal states in momentum space can be rewritten in the matrix form:

(A2) $$\begin{array}{c} H_{\mu \sigma ,\mu^{\prime }\sigma^{\prime }}\left(k\right)=\sum_{d}\exp\left(ik\cdot d\right)t_{R\mu \sigma ,R^{\prime }\mu^{\prime }\sigma^{\prime }}, \end{array}$$

where $d=R-R^{\prime }$ is the real-space distance between orbital $O_{1}$ and $O_{2}$. The band structure for a given k-point can be found by diagonalization of the matrix H(k).

In our case, the tight binding model was found from the 8×8×6 full k-point DFT calculation, starting from *p* orbitals of Sb, as well as *d* orbitals of Ag and La. This gives us 32-orbital tight binding model of the LaAgSb${}_{2}$. Comparison of the band structure obtained from the DFT and tight binding model can be found in Figure A1, while the Wannier (maximally localized) orbitals are presented in Figure A2.
