# Supplementary material for: Electronic Band Structure and Surface States in Dirac Semimetal LaAgSb2
Source: Materials (Basel). 2022 Oct 14;15(20):7168. doi: 10.3390/ma15207168 (PMC9609572; doi:10.3390/ma15207168)
Supplement: Supplementary file 1 [file materials-15-07168-s001.zip › materials-1943473-supplementary.pdf]

Supplemental Material: Electronic band structure and surface states in Dirac semimetal $LaAgSb_2$

Marcin Rosmus,[1,2] Natalia Olszowska,[2] Zbigniew Bukowski[3], Paweł Starowicz[1], Przemysław Piekarz[4], Andrzej Ptok[4]

[1] *Marian Smoluchowski Institute of Physics, Jagiellonian University, Prof. S. Łojasiewicza 11, PL-30348 Kraków, Poland*
[2] *Solaris National Synchrotron Radiation Centre, Jagiellonian University, Czerwone Maki 98, 30-392 Kraków, Poland*
[3] *Insitute of Low Temperature and Structure Research, Polish Academy of Sciences, P.O. Box 1410,50-950 Wrocław, Poland*
[4] *Institute of Nuclear Physics, Polish Academy of Sciences, ul. W. E. Radzikowskiego 152, PL-31342 Kraków, Poland*

(Dated: September 13, 2022)

In this Supplemental Material we present additional band structures obtained from DFT calculations, in particular:

- Figure S1 – the bulk electronic band structure related to layers forming $LaAgSb_2$ structure.
- Figure S2 – the Dirac points at X and M.
- Figure S3 – nodal lines formed along X–R line.
- Figure S4 – Fermi surface branches shown separately.
- Figure S5 – comparison of the band structures in $P4/mmm$ (*not realized in reality*, SG:123) and $P4/nmm$ (SG:129) showing impact of the mirror symmetry and glide symmetry.
- Figure S6–the bulk dectronic band structure along Γ–M path for $k_z = 0$ and $k_z = \pi/c$.

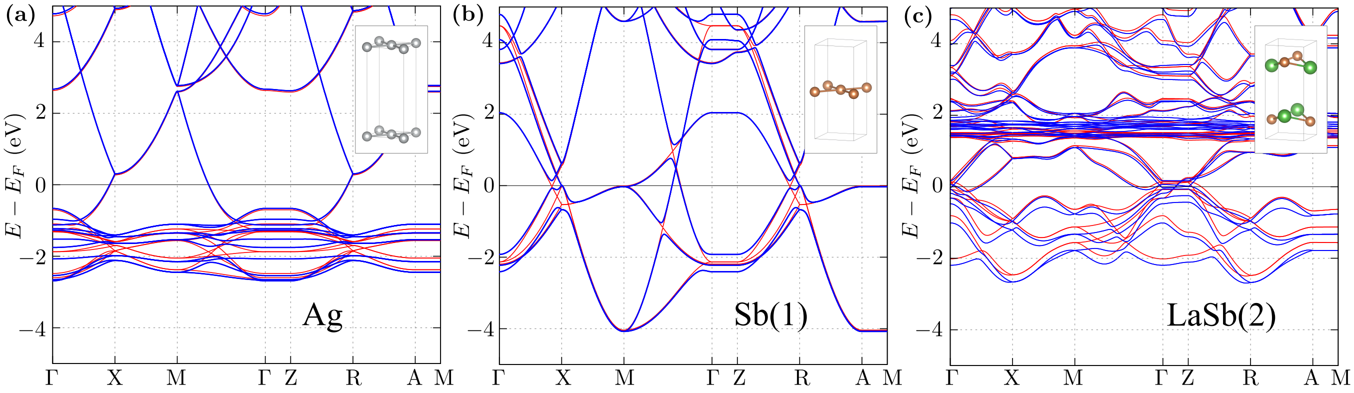

Figure S1. The bulk electronic band structure of layers forming bulk $LaAgSb_2$ structure (as labeled and shown in insets). Bands crossing the Fermi level in the case of Ag and Sb nets, correspond mostly to the p orbitals – which lead to the characteristic diamond–like Fermi surface with corner at X points. The Fermi surface centered at Γ is associated with bands of $LaSb_2$ double-layer showing nonsymmorphic glide symmetry.

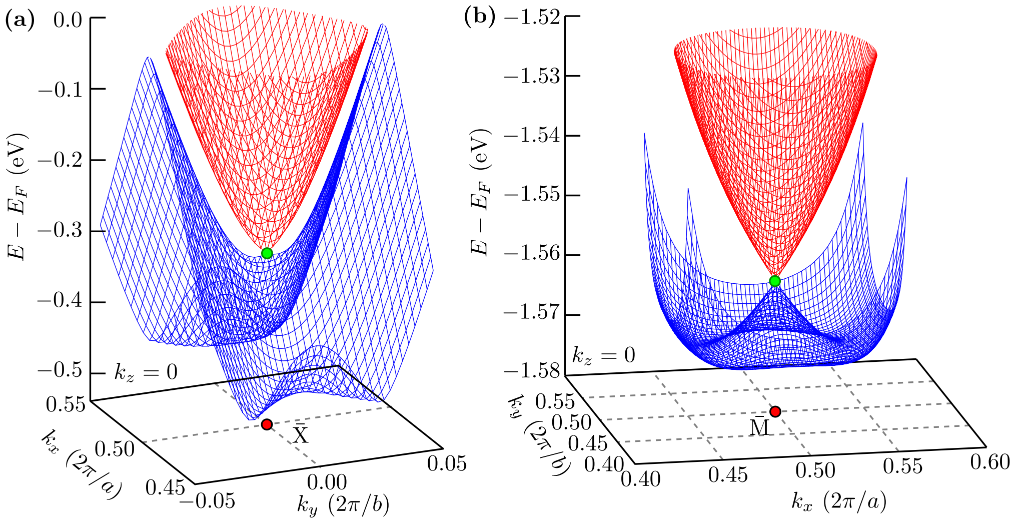

Figure S2. A realization of a Dirac point (green dot) at X (a) and M (b) points (zoom on insets at Fig. 2) forming a nodal line along X–A and M–R directions, respectively. In the case of the X point (a), the Dirac point is formed between the bottom vertex of the upper band (red) and the saddle point of the lower band (blue). In the case of M point (b), the Dirac point is realized between two bands a spliting of which is given by the spin–orbit coupling in the typical Rashba-like form.

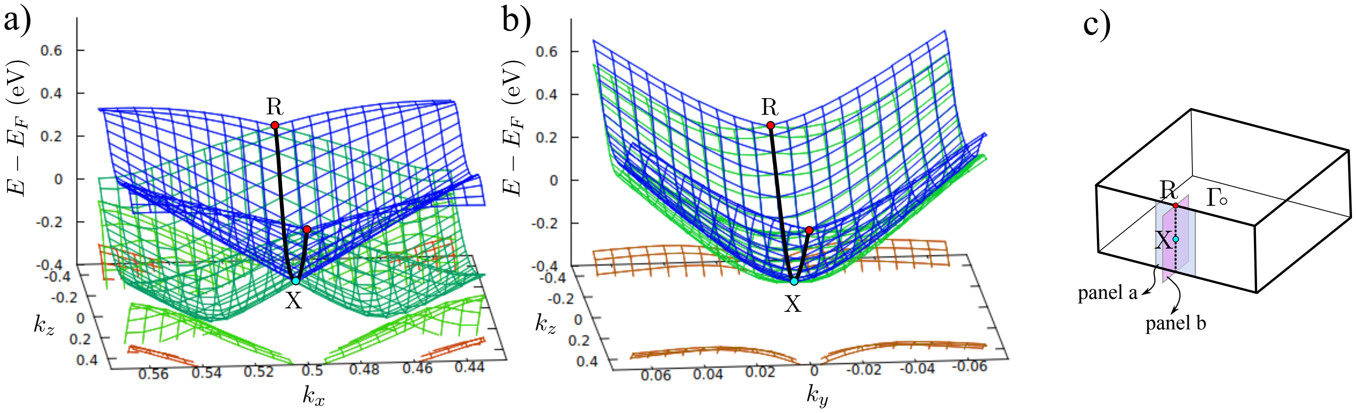

Figure S3. Band structures and Dirac nodal line along X–R direction. Panels (a) and (b) present dispersion relations in the vicinity of X–R direction along x and y axis, respectively [**k**-planes with respect to the Brillouin zone are presented in panel (c)]. Blue and red dots represent positions of X and R high symmetry points, respectively.

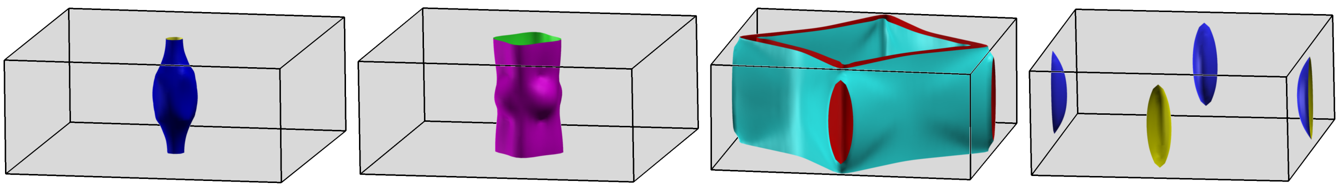

Figure S4. Branches of the Fermi surface of $LaAgSb_2$ shown in separate figures.

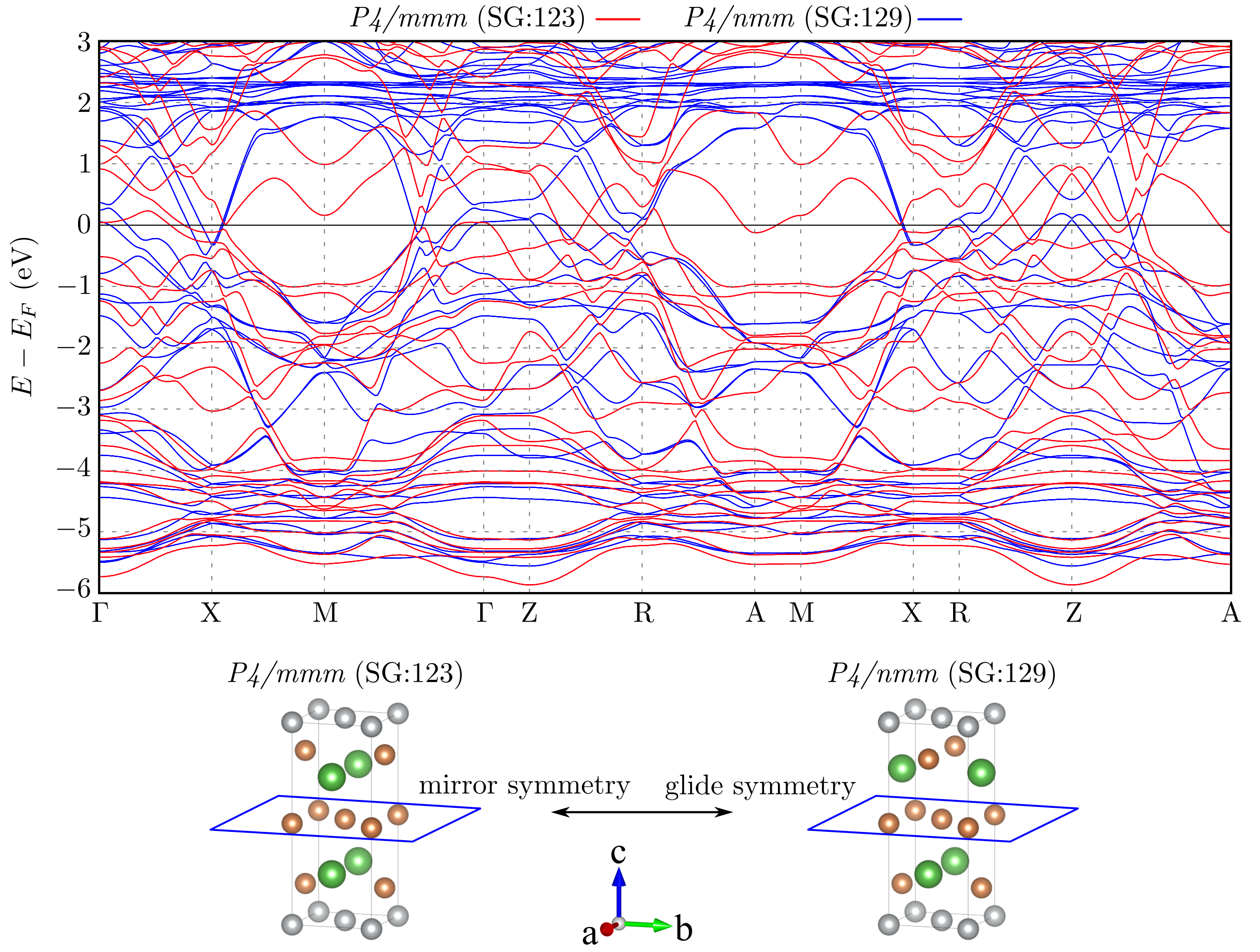

Figure S5. Comparison of the bulk electronic band structure of $LaAgSb_2$ for different structural variants, obtained from DFT calculations in the presence of the spin–orbit coupling. Red and blue lines correspond to structure with $P4/mmm$ and $P4/nmm$ space group, respectively. Replacement of the glide symmetry with the mirror symmetry (blue frame) causes a strong modification of the band structure of the system. In practice, this leads to a disappearance of the Dirac cones at high symmetry points X, M, R, or A, as well as a removal of degeneracy along A–M and X–R paths.

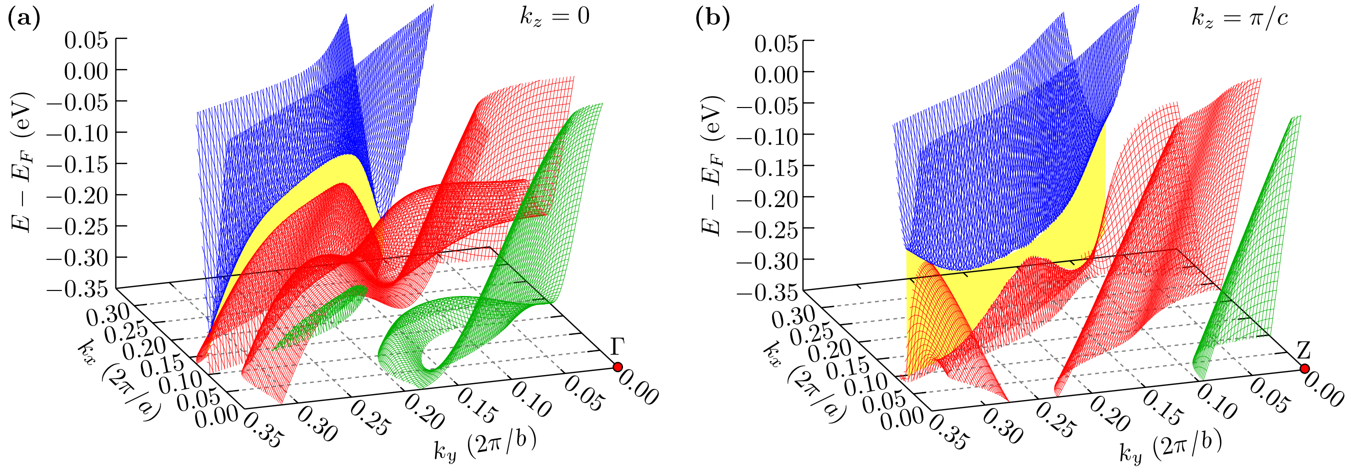

Figure S6. The bulk electronic band structure in the presence of the spin–orbit coupling along Γ–M path, for $k_z = 0$ (a) and π/c (b). The blue, red, and green surfaces correspond to the bands, while the yellow area shows the gap induced by spin–orbit coupling.
